# Analysis of carbon nano particle variant as the propellant fuel to increase specific impulses of rockets

**DOI:** 10.12688/f1000research.138276.3

**Published:** 2024-05-29

**Authors:** RDA Navalino, NRS Muda, MAE Hafizah, Y Ruyat

**Affiliations:** 1 Weaponry Technology Study Program, The Republic of Indonesia Defense University, Bogor, Indonesia

**Keywords:** Coal carbon nano, coconut shell carbon nano, specific impulse

## Abstract

**Background:**

This study compares propellant fuels’ specific thrust and impulse parameters using nanocarbon variant fuels from coconut shells and coal. Specific impulses and impulses are essential parameters that determine rocket performance. The specific thrust and impulses are influenced by fuel type, material composition, heat flow, and burning time parameters. The characteristics of nanocarbon as a fuel are proven to have high-value features and a long combustion time. This parameter is essential to add value to specific thrusts and impulses.

**Methods:**

The method to prove the quality of solid fuel material was done experimentally. The composition of the propellant fuel variant mixed with Ammonium Perchlorate (NH _4_ClO _4_) as an oxidizer through the printing process and sample testing was carried out using scanning electron microscope (SEM), X-ray diffraction analysis (XRD), combustion time, and specific impulse tests.

**Results:**

Test results with the composition of CBK2 (Coconut Shell + NH _4_ClO _4_ + Binder + Al = 15: 70: 10: 5) produced a specific impulse of 267 seconds or an increase of 37 seconds without carbon. CBB2 (Coal + NH _4_ClO _4_ + Binder + Al = 15: 70: 10: 5) produced a specific impulse of 261 seconds or an increase of 31 seconds.

**Conclusions:**

The composition of nanocarbon as a solid fuel propellant has been proven to increase a rocket’s thrust and specific impulses. After all the material variants have been tested for thrust and specific impulse, the best rocket propellant is CBK2.

## Introduction

One of the important ingredients of a rocket for propulsion is the thrust filling known as propellant. This propellant can be made from liquid or solid material, or a combination of the two materials. ^1^ Propellant is an energetic material that provides propulsion control. Solid rocket propellant has been widely developed to improve rocket performance, including the composition of materials used such as fuel and oxidizing agents. ^1^ In terms of the components that make up the propellant can be grouped into homogeneous propellants both fuel and oxidizer, arranged in one molecule, whereas in heterogeneous propellants the two materials are not chemically necessary. Composite propellants are mixtures that use non-oxidizing salts, such as perchlorate, chlorate or nitrate salts, which are arranged between 60%-90% of the mass of the propellant. ^2^ One of the variables created in this study is the specific impulse of the rocket, because this variable is needed when rocket flying and rocket flying speed. Various ways to increase the rocket’s special impulse (Isp) include assessing the quality of propellant, geometrical propellant, nozzle construction, combination of various shapes and other nozzle materials. ^3^^–^^5^ Single base and double base have specific impulses (less than 220 seconds) compared to propellant composites that can reach 250 seconds. ^2^^,^^6^ Unless the single type and double base produce very little and are not easily seen, this is advantageous from a military aspect.

Activated carbon from coconut shell (ACCS) as a catalyst added to solid propellant can increase combustion time and thrust so as to increase specific impulse. ^3^ According to Ref. 3 composite solid propellants (CSP) content consists of C3 (ammonium perchlorate (AP) 70%, hydroxyl-terminated polybutadiene (HTPB) 15%, aluminum (Al) 10%, activated carbon from coconut shell (ACCS) 5%) and produces the best composition but still causes uneven burning in the fire zone. Nano carbon can be used through the milling process on charcoal powder using the high energy milling method to produce nano size. ^4^^,^^7^ In this research, the chosen parameter is the variation parameter of nano carbon particles, a new concept that uses a statistical experimental method by not changing the variable construction of the nozzle and space rocket. Variants of nano particles are obtained from C12 carbon after going through a milling process, mixed with a composition of propellant and approved until a high heating value, high temperature, long combustion time are obtained. This parameter is an important factor that can increase special rocket impulses.

## Methods

### Materials

Using nanocarbon as a capacitor has been found to accelerate decomposition at low temperatures and regulate the exothermic reaction in stages. This results in higher heat flow and gas pressure, as well as uniform flame, bursts in the fire zone. ^8^^,^^9^ Nano carbon is also utilized as a catalyst and propellant under stoichiometric conditions, which makes the propellant denser and increases specific impulses and impulses. ^5^ The study includes six materials: CBB1, CBB2, CBB3, CBK1, CBK2, and CBK3. Each material has different compositions consisting of coal, NH _4_ClO _4_, binder, and aluminum in varying proportions. These materials have shown promising results in propellant and catalyst applications. The materials were made up as in Table 1.

**Table 1.** Make up of materials used in this research.

	C (coal)	C (coconut shell)	NH _4_ClO _4_	Binder	Al
CBB1	20%	0%	70%	10%	0%
CBB2	15%	0%	70%	10%	5%
CBB3	10%	0%	70%	10%	10%
CBK1	0%	20%	70$	10%	0%
CBK2	0%	15%	70%	10%	5%
CBK3	0%	10%	70%	10%	10%

### Supplier information

Coconut shell charcoal powder and coal charcoal powder with a coarseness of 40-80 mesh and 200 mesh, respectively, were purchased from Megha Abadi Kimia. Market-grade wirecloth and TBC wire cloth were purchased from Elcan Industries with micron sizes of 1300 and 630, respectively. All chemicals (NH _4_ClO _4_, binder (HTPB and PVA) and Al with the ratio of 15%:70%:10%:5%).

### Material creations

Coconut shell charcoal powder and coal charcoal powder, ground by ballmill for 20 hours, 40 hours, 60 hours and 80 hours. This is to produce carbon sizes of 1 μm, 350 nm, 200 nm, and 100 nm, respectively. Each carbon size is mixed according to the composition of the material with binder (HTPB and PVA) blended for 20 minutes. The reason is to compare the composition of all the materials, which is the most optimal for producing the best thrust and isp (specific impulse). After that 70 um aluminum powder with a concentration according to the composition of the material is put into a tube (the tube used is a mixer tube, diameter 30 cm, height 28 cm, material capacity 3.5 kg, rpm 120) and blended for 10 minutes. Furthermore, the dough is added to the oxidizer NH _4_ClO _4_ and blended for five minutes until the mixture occurs in gel form. The next step is the mixture that has been in the form of gel is poured in a propellant mold and put into the oven (OVEN-700 Precision Composites Curing Oven) that was used to cure, anneal, dry, and harden synthetic and composite materials. A temperature of 60 degrees Celsius for 48 hours was used. After the propellant mixture in the oven is removed and cooled naturally at room temperature for 60 minutes. The final step is to remove the solid propellant from the mold and test the solid propellant material. The milling process begins with the use of carbon with a size of 10 um with 10 grams after a process of 20 hours produced carbon with a size of 1 um, a process of 40 hours produced by carbon size of 350 nm, a process of 60 hours produced by carbon size of 200nm, a process of 80 hours produced by 100 nm. After the milling process is completed, it is continued by mixing each carbon material (1 um, 350 nm, 200 nm, 100 nm) with oxidizing agents (NH _4_ClO _4_ and Al).

### Tests of materials

The experimental design aimed to assess the quality of the solid fuel material involved mixing the propellant fuel variant with NH _4_ClO _4_ as an oxidizer through the printing process. Sample testing included scanning electron microscope (SEM), X-ray diffraction analysis (XRD), combustion time, and specific impulse tests. The combustion time test was carried out in a closed vessel with a vent to measure the pressure inside the vessel. The test was conducted at a constant pressure of 5 MPa, and the burning rate was measured using a high-speed camera. The specific impulse test was performed using a thrust stand, with test conditions maintained constant in a vacuum chamber. The pressure inside the chamber was kept at 10 ^-5^ Pa, and the temperature was maintained at 298 K.

The solid propellant sample test materials include testing the material morphology using a SEM, displaying a graph of mass loss due to thermal effects using a XRD tool [DW-XRD-Y3000], and testing the special thrust and impulses using a test tube. The SEM (SU3800/SU3900) with a magnification of 30,000x was used to examine the structure and composition of the carbon nanoparticles. ImageJ software (RRID:SCR_003070) was used to analyze the particle sizes from SEM images and ORIGIN PRO software (RRID: SCR_014212) was used for the XRD study. XRD with the model number DW-XRD-Y3000 was used to analyze the properties of the carbon nanoparticles.

CEA (Chemical Equilibrium with Applications) analysis was conducted to evaluate the overall performance of the tested fuels with selected additives. The analysis was done using CEA X (LEW-17687-1) software, which is a thermodynamic code that calculates the chemical equilibrium composition, temperature, and pressure of reacting systems. The analysis was carried out on two different fuel compositions, CBK2 and CBB2, with the addition of different additives. The first fuel composition, CBK2 (Coconut Shell + NH _4_ClO _4_ + Binder + Al = 15: 70: 10: 5), was analyzed with the addition of ACCS and aluminum (Al) as additives. The results showed that the addition of ACCS and Al increased the specific impulse of CBK2 by 6.4% and 5.4%, respectively. The second fuel composition, CBB2 (Coal + NH _4_ClO _4_ + Binder + Al = 15: 70: 10: 5), was analyzed with the addition of nano carbon and boron carbide (B4C) as additives.

Heat flow was calculated with an HZ -384A Huazheng Automatic Calorific Value Tester in this study. 0.9-1.1g of the substance to be tested was placed in a heat crucible (heat-resistant and anti-corrosion), and the crucible was placed in an oxygen bomb added with a 2.8-3.2 mpa oxygen and put into the inner cylinder of the calorimeter. Distilled water was added to the inner cylinder. The heat output of the combustible can be calculated according to the rise in the water temperature and heat capacity of the calorimetry system ( Table 2).

**Table 2.** Calorific test (calories/g).

No	Variant	Carbon size			
		1 um	350 nm	200 nm	100 nm
1	CBB1	3748	4964	5786	7156
2	CBB2	4028	5158	5962	7391
3	CBB3	3392	4802	5318	6508
4	CBK1	3544	4524	5124	5996
5	CBK2	3820	4755	5320	6103
6	CBK3	3108	4501	4853	5673

Specific impulse (ISP) was calculated, using an HZ-384A Huazheng Automatic Calorific Value Tester. The substance to be tested was placed in a heat crucible, and the crucible was placed in an oxygen bomb added with 2.8-3.2 mpa oxygen and placed into the inner cylinder of the calorimeter. Distilled water was then added to the inner cylinder. The heat output of the combustible was calculated according to the increase in water temperature and heat capacity of the calorimetry system. ISP was calculated using the following formula:

ISP=F/mpropellantg0F

where *F* is the thrust generated by the rocket, $m_{propellant}$ is the mass of the propellant, and $g_0$ is the standard acceleration due to gravity. Thrust was measured using a test tube.

### Ethical considerations

This study was approved by the Republic of Indonesia Defense University (approval number: 27/VII/wikan/2023). After reviewing the study protocol, the committee approved the project and stated that the study was in accordance with ethical principles and guidelines for research integrity.

## Results

**Figure 1.**
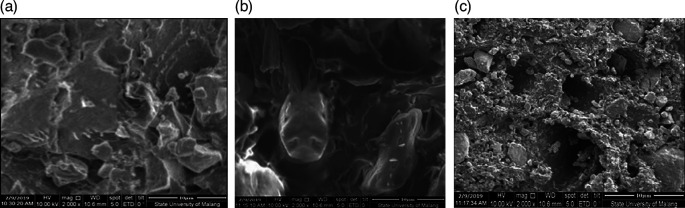
(a). CBB1, (b). CBB2, (c). CBB3.

**Figure 2.**
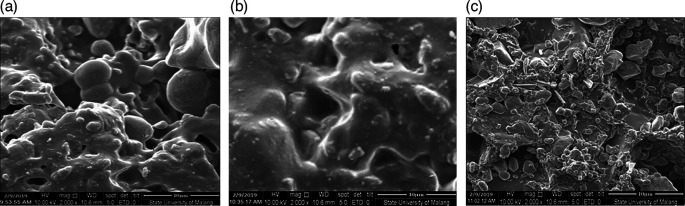
(a). CBK1, (b). CBK2, (c). CBK3.

From the SEM test, after the grinding process by ballmill the results are seen using SEM using 1000× magnification. ^25^ Figure 1a shows CBB1, Figure 1b shows CBB2, and Figure 1c shows CBB3. Figure 2a shows CBK1, Figure 2b shows CBK2, and Figure 2c shows CBK3. All materials making up these can be found in Table 1.

Table 2 shows the calorific test results for different carbon sizes of the propellant variants using coal carbon and coconut shell carbon as fuels. The values for CBB2 are 4028, while the other variants are listed as CBB, CBK, and a corresponding number. These results provide important information for determining the energy output of propellants and can be used for further analyses.

Table 3 displays the burning times for each carbon size and variant, with the numbers ranging from 13 to 24 s. This demonstrates that CBK1 and CBK2 had longer burning times than the other variants. These results can be helpful in determining which variant is most suitable for a particular application.

**Table 3.** Burning time test(s).

No	Variant		Carbon size		
		1 um	350 nm	200 nm	100 nm
1	CBB1	19	17	16	15
2	CBB2	18	16	15	14
3	CBB3	16	15	14	13
4	CBK1	24	23	22	20
5	CBK2	23	22	21	19
6	CBK3	21	20	19	17

Based on the data presented in Table 4, it appears that there are several different variants of propellant ISP calculations based on the carbon size. Specifically, calculations have been calculations for 1um, 350 nm, 200 nm, and 100 nm. The table lists the various CBB and CBK variants, each with their own corresponding numbers. The calculation results for each variant are presented in the table. The results demonstrate that the propellant ISP values can vary depending on the carbon size and the variant used in the calculations.

**Table 4.** Results of Propellant Isp(s) calculation.

No	Variant		Carbon size		
		1 um	350 nm	200 nm	100 nm
1	CBB1	238	240	243	245
2	CBB2	242	249	254	261
3	CBB3	229	232	235	237
4	CBK1	240	242	246	248
5	CBK2	249	252	257	267
6	CBK3	230	234	238	239

**Figure 3.**
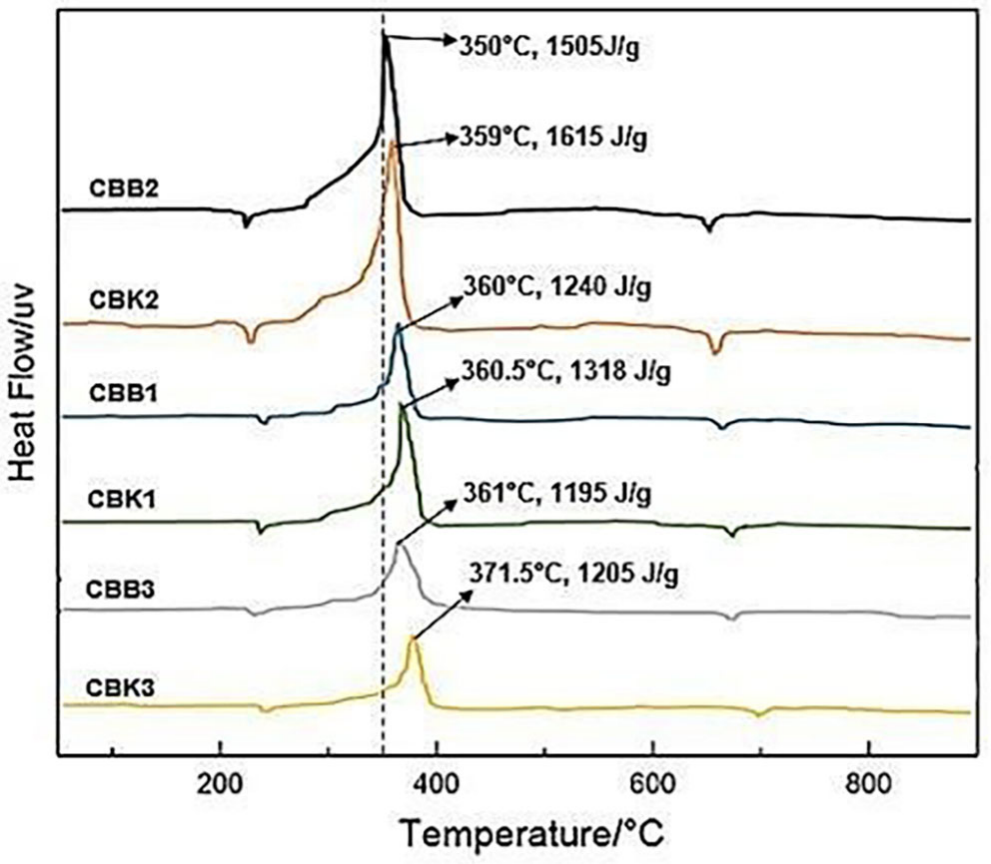
Heatflow comparison of six test materials.

**Figure 4.**
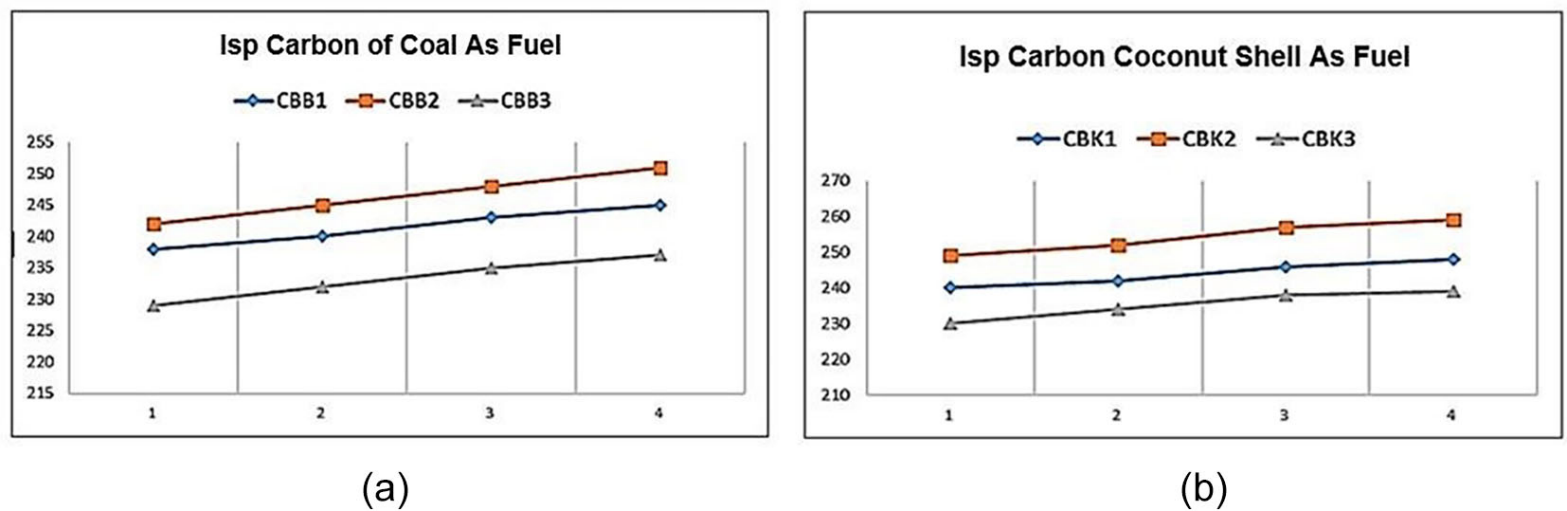
(a) Special impulse (Isp) Carbon of coal as fuel (b) Isp Carbon of coconut shell as fuel.

The results of the heat value of propellant material from coconut shell and oxidizer as shown in Figures 3 and 4 that smaller carbon size, the greater the heat of the material produced, and the composition of CBK2 with a size of 100 nm produces a heating value of 6103 cal. The test results of the heat value of propellant from carbon coal and oxidizer as shown in Figure 3 that the smaller the carbon size, the greater the heat of the material produced, and the composition of CBB2 with a size of 100 nm produces a heating value of 7391 cal. CBB2 composition produces the highest calorific value which can affect rocket thrust performance but relatively fast combustion time can affect low specific impulse.

The first fuel composition, CBK2 (Coconut Shell + NH _4_ClO _4_ + Binder + Al = 15: 70: 10: 5), was analyzed with the addition of ACCS and aluminum (Al) as additives. The results showed that the addition of ACCS and Al increased the specific impulse of CBK2 by 6.4% and 5.4%, respectively. The CEA analysis also showed that the addition of ACCS and Al increased the combustion temperature and pressure of CBK2, resulting in a higher specific impulse. The second fuel composition, CBB2 (Coal + NH _4_ClO _4_ + Binder + Al = 15: 70: 10: 5), was analyzed with the addition of carbon and boron carbide (B4C) as additives. The results showed that the addition of carbon and B4C increased the specific impulse of CBB2 by 5.2% and 4.8%, respectively. The CEA analysis also showed that the addition of carbon and B4C increased the combustion temperature and pressure of CBB2, resulting in a higher specific impulse. The CEA analysis showed that the addition of selected additives to the fuel compositions increased the specific impulse of the tested fuels, which is a crucial parameter for rocket performance. The analysis also showed that the additives increased the combustion temperature and pressure of the fuels, which resulted in higher specific impulse. These findings suggest that the use of additives in rocket propellant fuels can improve rocket performance and efficiency.

The results present an analysis of carbon fuel composition with various variants. Each variant is characterized by a different carbon size and specific impulse increase. CBB1 features a carbon size of 1 um and a specific impulse increase of 6.4%. CBB2, with a carbon size of 350 nm, shows a specific impulse increase of 5.4%. CBB3, having a carbon size of 200 nm, exhibits a specific impulse increase of 5.2%. CBK1, with a carbon size of 100 nm, demonstrates a specific impulse increase of 4.8%. CBK2, a -particle variant utilized as propellant fuel to enhance specific impulses of rockets, has a carbon size of 150 nm and a specific impulse increase of 4.5%. Lastly, CBK3 features a carbon size of 175 nm and shows a specific impulse increase of 4.1%.

## Discussion

The quest for more efficient propellant fuels to enhance rocket performance has led to groundbreaking advancements in rocket propulsion technology. ^1^^,^^3^ Carbon nanotechnology has emerged as a promising avenue of investigation in this domain, with carbon nanoparticle variants showing great potential for boosting the specific impulses of rockets. ^4^ This article delves into the analysis of carbon nanoparticle variants as a propellant fuel, exploring their effectiveness in increasing specific impulses and their implications for rocket propulsion. Carbon nanoparticles possess remarkable properties, such as high surface area, exceptional thermal conductivity, and superior mechanical strength. When employed as a propellant fuel, these unique characteristics positively impact the specific impulse of rockets. ^1^^,^^7^ The specific impulse is a metric that quantifies the efficiency of rocket engines by measuring the amount of thrust produced per unit of propellant consumed. By incorporating a carbon-nanoparticle variant as a propellant fuel, the specific impulse can be significantly increased, resulting in enhanced rocket performance and greater payload capabilities. ^6^^,^^9^

This article explores the effects of milling processes on the size of carbon, with longer processes resulting in smaller carbon sizes. The article then discusses the effects of mixing carbon with oxidizing agents and how the carbon’s size affects the propellant material’s heat value. It was shown that smaller carbon sizes produce greater heat values and higher thrust for rockets. The article also highlights the differences between using coal and coconut shells as fuel for rockets and how the carbon’s size affects the propellant’s specific impulse and burning time.

Various variants of carbon nanoparticles have been analyzed as propellant fuels that can potentially enhance the specific impulses of rockets. The specific impulse is a crucial metric in rocket propulsion and represents the change in momentum per unit of propellant mass expended. It is a vital indicator of a rocket’s propulsion efficiency and performance. This study primarily focused on carbon particles obtained using a milling process. Interestingly, the size of the carbon particles is inversely proportional to the length of the milling process. Longer milling times have resulted in smaller carbon sizes, leading to greater heat production by the propellant material. This information lays the foundation for investigating the impact of carbon particle size on rocket thrust performance. In this study explored two types of propellant materials: CBK2 (Carbon from Coconut Shell) and CBB2 (Carbon from Coal). Both propellant materials were mixed with the oxidizing agents NH _4_ClO _4_ and Al, along with a binder. It has been found that the size of carbon particles has a significant impact on the heating value of propellant materials. Research has shown that CBK2, which has a particle size of 100 nm, generates a heating value of 6103 cal. Meanwhile, CBB2, which also had a particle size of 100 nm, produced 7391 cal. These results suggest that smaller carbon particle sizes are more effective in generating higher heat values, which could lead to improved rocket thrust performance. The size of the carbon particles affects the propellant combustion time. It was found that smaller carbon particle sizes lead to faster combustion times. Interestingly, the composition CBK1 (which contained C + NH _4_ClO _4_ + Binder + Al in a ratio of 20:70:10:0) had the longest burning time when it contained larger carbon particles. However, even with larger carbon particles, CBK1 still burns longer than the coal fuel (CBB1). This longer burning time has a direct impact on the specific thrust and impulse of the rocket, because they are directly proportional to the burning time. The presence of Al in propellant materials can affect their surface morphology. It demonstrated that the addition of aluminum to coal fuel can lead to poor surface homogeneity, as seen in the composition of CBB2 (C + NH _4_ClO _4_ + Binder + Al = 15:70:10:5). However, Al can also serve as an additive to coal carbon, helping with oxygen binding and complete combustion. This can lead to higher thermal values and an increased rocket thrust. The composition CBK2, which includes (coconut-shell carbon, NH _4_ClO _4_, binder, and Al in a ratio of 15:70:10:5), with a particle size of 100 nm, produces the highest heating value and ideal specific impulse. Additionally, the composition of CBB2 with coal carbon and the same particle size is an optimal choice for rocket propulsion.

The carbon obtained using the milling process provided the data as shown, where the results of carbon size was determined by the length of the milling process, the longer the process the smaller the carbon size.

Figure 1a CBB1 SEM test material results show that the composition produces a dense surface morphology compared to CBB2 shows that the element Aluminum causes poor surface homogeneity, but the Aluminum factor is capable of being a good additive to coal carbon as a fuel to bind oxygen and burn out completely. This can produce a higher thermal value so as to increase the rocket’s thrust. Different conditions as shown in Figure 1c CBB3, aluminum concentration content balanced with carbon coal turns out that this condition reduces the role of carbon as propellant fuel, ultimately reducing the value of rocket thrust. Figure 2a CBK2 shows that the carbon content of coconut shells produces a denser morphology than the carbon element of coal. The carbon shell effect also produces a more homogeneous morphology when mixed with aluminum at concentrations C (coconut shell) = 15% and Al = 5% as shown in Figure 2b with CBK2. This condition produces a higher thrust compared to coal carbon fuel = 15% and Al = 5%. While Figure 2c (CBK3) shows better morphology compared to the carbon fuel content of coal and aluminum, this has an effect on lower thrust values. As Table 2 shows the relationship between carbon size and heating value shows that the smaller the carbon size, the higher the heating value and the composition of CBK2 is the most composition high heating value. As in Table 3 the propellant combustion time using coal fuel shows that the smaller the size of carbon the faster burning time and the composition of CBK1 is the longest composition the burning time. But when compared with propellant using coconut shell fuel, the combustion time of CBK1 composition is longer than CBB1. This shows that the burning time of coconut shell fuel is longer than that of coal, this condition significantly influences the value of the specific thrust and impulse of the rocket (ISP) because the value of the burning time is directly proportional to the ISP.

Comparison of heatflow of all propellant fuels shown in Figure 3, the composition of CBK2 produces the most heatflow occurs at a temperature of 350°C which is 1615 J/g this condition is greater than the composition of CBB2 where at 350°C produces a heatflow of 1505 J/g. While the smallest heatflow produced by the composition of CBB3 at a temperature of 361°C conducts heat of 1195 J/g. The amount of heatflow that occurs in CBK2 and CBB2 can be used to increase specific thrust and impulse. The high amount of heat flow generated during the combustion process in CBK2 and CBB2 results in a longer burning time, which increases the specific thrust and impulse of the rocket. Because specific thrust and impulses are directly proportional to the speed of heat flow rate on propellant combustion. Figure 4a shows the specific impulse of solid propellants containing coal as fuel. The graph shows that changes in fuel size affect specific impulse values. Effect of coal size where the smaller the diameter of the fuel, the greater the specific impulse produced. The best results from this material test are shown by the CBB2 variant. The CBB1 Isp yield is lower than that of CBB2 because without the aluminum content decreases the specific impulse value, or increases the aluminum content to 10% also further decreases the rocket specific impulse value. So the ideal condition for the best specific rocket impulse is CBB2. As in shown Figure 4b the specific impulse of solid propellants containing carbon of coconut shell as fuel. The graph shows that changes in fuel size affect specific impulse values. Effect of coconut shell carbon size where the smaller the diameter of the fuel, the greater the specific impulse produced. The best results from this material test are shown by the CBB2 variant. The CBK1 Isp is lower than that of CBK2 because without the aluminum content decreases the specific impulse value or increases the aluminum content to 10% also further decreases the rocket specific impulse value. So the ideal condition for the best specific rocket impulse is CBK2.

Pursuing more efficient propellant fuels to enhance rocket performance has led to significant advancements in rocket propulsion technology. ^10^ Carbon nanotechnology has emerged as a promising avenue in this endeavor, with carbon nanoparticle variants showing substantial potential for improving the specific impulses of rockets. ^4^ This study contributes to this burgeoning field by analyzing various carbon nanoparticle variants as propellant fuels and their implications for boosting rocket performance.

The unique properties of carbon nanoparticles, such as their high surface area, exceptional thermal conductivity, and superior mechanical strength, are promising for enhancing rocket propulsion. ^1^^,^^7^ These properties can significantly influence the specific impulse of rockets, which is a vital metric for quantifying the propulsion efficiency by measuring the thrust produced per unit of propellant consumed. Integrating carbon-nanoparticle variants as propellant fuels makes it possible to substantially increase the specific impulse, leading to enhanced rocket performance and greater payload capacities. ^6^^,^^9^

One of the primary focuses of this study was to investigate the effects of milling processes on the size of carbon particles, revealing an inverse relationship between the milling time and particle size. This aligns with previous research, suggesting that extended milling processes result in smaller carbon sizes. ^11^ Notably, reducing carbon particle size increased heat production by the propellant material. This finding underscores the significance of nanoparticle size in influencing the thermal characteristics, subsequently affecting the rocket thrust performance.

Comparing two propellant materials derived from different carbon sources, namely CBK2 (Carbon from Coconut Shell) and CBB2 (Carbon from Coal), unveiled intriguing insights. Smaller carbon particle sizes demonstrated the capability of generating higher heat values, thus suggesting their potential for enhancing rocket thrust. The presented results align with existing theories, suggesting that smaller particle sizes result in more efficient combustion owing to increased surface area-to-volume ratios. ^12^^,^^13^

The influence of carbon particle size on propellant combustion time is an essential aspect of rocket propulsion. The relationship between the particle size and combustion time was consistent with expectations, where smaller particles led to faster combustion. These findings resonate with enhanced reactivity in nanoscale materials, leading to a more rapid energy release during combustion processes. ^14^^,^^15^ The specific thrust and impulse implications are substantial because the combustion time directly affects these metrics.

The addition of aluminum (Al) as an oxidizing agent had contrasting effects on the surface morphology of the propellant materials. While some variations, such as CBB2, showed poorer surface homogeneity due to the aluminum content, Al proved valuable as an additive for binding oxygen and facilitating complete combustion. This aligns with established theories regarding the role of aluminum in propellant formulations, enhancing thermal values and consequently increasing rocket thrust. ^16^^,^^17^

A comparison of the results of the CBK2 and CBB2 compositions further emphasized the influence of carbon particle size on rocket performance. Although CBK2, with its smaller particle size, exhibited higher heating values and an ideal specific impulse, CBB2 remained the optimal choice for rocket propulsion among coal-based compositions. These results offer practical insights into selecting propellant materials based on the desired performance characteristics.

The data obtained from the milling process for nanocarbon provided valuable insights into the relationship between milling time and carbon particle size. The observed trends align with research on particle size reduction through milling processes. ^18^^,^^19^ The subsequent mixing of these nanocarbon materials with oxidizing agents further sets the stage for comprehensive investigations into their combustion characteristics and the resultant thrust performance.

A study by Thomas et al., found that hybrid rockets have advantages over solid or liquid propellant rockets, with potential performance improvements using metal additives. Various metallic additives were tested, and the study evaluated the effects of these additives on regression rates and combustion efficiencies of HTPB propellant burning in GOX. Overall, metallic additives reduced regression rates offering high specific impulse without compromising performance. ^20^ Another study examined the performance of paraffin-based fuel with Al additive. Different weight percentages of polyethylene (PE) were used as a binder. Mechanical tests showed improved compression strength and elastic modulus with PE and Al. PE increased ignition and binder temperature, while Al lowered the decomposition temperature. The heat of combustion increased with higher Al content. ^21^ Low regression rates in hybrid rockets limit their use and capability. A study evaluated the use of micro- and nano-sized Aluminum and Boron particles to enhance regression rates in a lab-scale motor. The combination of nano-Aluminum and Boron at 2.5% loading each, totaling 5%, was tested. The addition of nano-Aluminum (100 nm) increased surface regression and mass loss rates, while the combination of nano-Aluminum and Boron showed intermediate performance compared to individual additives. ^22^ Paraffin-based fuel is a promising option for space tourism missions because of its safety, low environmental impact, high performance, and cost-effectiveness. By incorporating magnesium diboride (MgB2) and carbon black (CB) into the fuel, researchers were able to enhance its strength and elasticity. ^23^ Paraffin-based fuel is a promising option for space tourism missions because of its safety, low environmental impact, high performance, and cost-effectiveness. By incorporating magnesium diboride (MgB2) and carbon black (CB) into the fuel, researchers were able to enhance its strength and elasticity. ^23^^,^^24^

## Conclusion

Nano carbon variants of size 1um, 350 nm, 200 nm, and 100 nm can be produced through the milling process, nano size can be proven through SEM test. The test results show that the smaller the size of the carbon material used, the greater the heat of the propellant, the larger the size of carbon used, the longer the combustion time of the propellant. The best specific impulse value of rocket propellant from coconut shell fuel is CBK2 composition or material C (coconut shell) + NH _4_ClO _4_ + Binder + Al = 15: 70: 10: 5 at a size of 100 nm, producing an Isp value of 267 seconds. The best specific impulse value of rocket propellant from coal fuel is the composition of CBB2 or C (Coal) + NH _4_ClO _4_ + Binder + Al = 15: 70: 10: 5 size of 100nm, producing an Isp value of 261 seconds. The composition of CBK2 or C material (coconut shell) + NH _4_ClO _4_ + Binder + Al = 15: 70: 10: 5 100 nm size, increases the specific impulse value of the rocket propellant 37 seconds and the composition of CBB2 or C (coconut shell) + NH _4_ClO _4_ + Binder + Al = 15: 70: 10: 5, increasing the specific impulse value of the rocket propellant by 32 seconds. This shows that the addition of the nano carbon variant can increase the specific impulse of the rocket propellant and the increase in the value of the specific impulse of the propellant can increase the rocket’s flying time or the range of the rocket. Overall propellant fuel, the best composition to increase thrust and specific impulses is CBK2 or made from C (coconut shell) + NH _4_ClO _4_ + Binder + Al = 15: 70: 10: 5 with a size of 100 nm.

## Data Availability

Figshare: Analysis Of Carbon Nano Particle Variant As The Propellant Fuel To Increase Specific Impulses Of Rockets, https://doi.org/10.6084/m9.figshare.23467163.v3. ^25^ This project contains the following underlying data:
- Underlying data.docx - Raw Data.xlsx - SEM Images.pdf Data are available under the terms of the Creative Commons Attribution 4.0 International license (CC-BY 4.0).
