# Supplementary material for: Analysis of carbon nano particle variant as the propellant fuel to increase specific impulses of rockets
Source: F1000Res. 2024 May 29;12:1414. Originally published 2023 Oct 26. [Version 3] doi: 10.12688/f1000research.138276.3 (PMC11350321; doi:10.12688/f1000research.138276.3)
Supplement: Peer review history

# Reviewer report: Reviewer response for version 3

**DOI:** 10.5256/f1000research.166219.r296166

**Published:** 2024-08-29

**Authors:** Sachin Srivastava

**Affiliations:** 1 Department of Aeronautical Engineering, MRCET, Hyderabad, Telangana, India

Specify the formulas, so that statistical values will justify.

 Without mentioned analytical work and assumptions, the statistical work is meaningless.

 This paper is about the propellant combustion characteristics such as specific Impulse and Thrust, constraint using additives nanocarbon variant fuels from coconut shells and coal. Combustion characteristics are essential to focused that determine rocket performance. These combustion characteristics, specific impulse and thrust are influenced by type of fuel, composition of material composition, heat flow, regression rate and burning time.

Is the work clearly and accurately presented and does it cite the current literature?

Yes

If applicable, is the statistical analysis and its interpretation appropriate?

Partly

Are all the source data underlying the results available to ensure full reproducibility?

Partly

Is the study design appropriate and is the work technically sound?

Yes

Are the conclusions drawn adequately supported by the results?

Yes

Are sufficient details of methods and analysis provided to allow replication by others?

Yes

Reviewer Expertise:

Fluid Mechanics, Thermodynamic, Hybrid Rocket Engine, Solid Rocket Motor, Liquid Rocket Engine, Scramjet Engine, Ramjet Engine

I confirm that I have read this submission and believe that I have an appropriate level of expertise to confirm that it is of an acceptable scientific standard.

# Reviewer report: Reviewer response for version 3

**DOI:** 10.5256/f1000research.166219.r296167

**Published:** 2024-08-27

**Authors:** Viatcheslav Naoumov

**Affiliations:** 1 Central Connecticut State University, New Britain, Connecticut, USA; 2 Engineering, Central Connecticut State University School of Engineering Science and Technology (Ringgold ID: 428313), New Britain, Connecticut, USA

Thank you for the opportunity to review the research paper “Analysis of carbon nano particle variant as the propellant fuel to increase specific impulses of rockets”

 In addition to my answers to your questions I would like to express some comments.

1. The paper introduces new results on the influence of the additives – nanoparticles on the parameters of solid fuels and could provide scientists and practitioners with important data which could be helpful for the design of new solid propellants.
2. I would recommend the proofreading to increase the readability of the article.
3. There are a lot of inaccuracies and mistakes in the terminology which unfortunately doesn’t correspond to the standard terminology employing in the area of rocketry/rocket propulsion and can confuse the reader. A few examples:

1. “Impulses” (the only parameter of the rocket engine related to the word “impulses”  is the “specific impulse” (not “impulses” and also not “special impulse” which the authors use as well);
2. In application to rocket engines the “specific thrust” is the same as “specific impulse”
3. Term “propellant fuel” is wrong since “fuel” it is exactly fuel, but propellant is a combination of fuel and oxidizer;
4. A lot of  phrases, sentences  and statements are not understandable both from the point of view of language and professional language which we use in rocketry area, such as:

“One of the important ingredients of a rocket for propulsion is the thrust filling known as propellant “ ?, “Propellant is an energetic material ….. oxidizing agents” ?; “…two materials are not chemically necessary” ?  (see Introduction), and much - much more !. It is confusing. It seems to me that paper requires the proof-reader – specialist in the rocketry/rocket engines.

1. The paper  is replete with repetitions of the same information and analysis in the sections “Results”, “Discussion” and “Conclusion”. The structure requires optimization.
2. I have some doubts regarding the values of the combustion temperature presented  in the Table 2. Temperatures which are higher than 4000 (doesn’t matter – in  K or C) and even higher than 7000 (as shown in the Table 2) are unbelievable for the combustion of such propellants. Even the “most high-energy” liquid propellant  “fluorine +hydrogen” gives about  5000K-6000K at stoichiometry.  The authors should check their thermodynamic calculations!
3. It looks like the authors made the mistake in the formula for the calculation of specific impulse. The correct formula is: Isp=Thrust/(mass flow rate of the propellant),  which gives Isp in seconds or meters/seconds depending on the units of thrust (kg or N). Author’s formula leads to the wrong units of the specific impulse.
4. Finally, the results presented in the paper are interesting and helpful to my opinion, but the paper requires corrections and improvements

Is the work clearly and accurately presented and does it cite the current literature?

No

If applicable, is the statistical analysis and its interpretation appropriate?

Not applicable

Are all the source data underlying the results available to ensure full reproducibility?

Partly

Is the study design appropriate and is the work technically sound?

Yes

Are the conclusions drawn adequately supported by the results?

Partly

Are sufficient details of methods and analysis provided to allow replication by others?

Partly

Reviewer Expertise:

Aerothermodynamics, Rocketry, Liquid, Solid and Hybrid Propellant Rocket Motors.

I confirm that I have read this submission and believe that I have an appropriate level of expertise to confirm that it is of an acceptable scientific standard, however I have significant reservations, as outlined above.

# Reviewer report: Reviewer response for version 3

**DOI:** 10.5256/f1000research.166219.r284356

**Published:** 2024-06-27

**Authors:** Syed Alay Hashim

**Affiliations:** 1 Alliance University, Bangalore, Karnataka, India

Now it seems fine.

Is the work clearly and accurately presented and does it cite the current literature?

Yes

If applicable, is the statistical analysis and its interpretation appropriate?

Partly

Are all the source data underlying the results available to ensure full reproducibility?

Partly

Is the study design appropriate and is the work technically sound?

Yes

Are the conclusions drawn adequately supported by the results?

Partly

Are sufficient details of methods and analysis provided to allow replication by others?

Yes

Reviewer Expertise:

Rockets, energetic materials, propulsion and combustion.

I confirm that I have read this submission and believe that I have an appropriate level of expertise to confirm that it is of an acceptable scientific standard.

# Reviewer report: Reviewer response for version 2

**DOI:** 10.5256/f1000research.159934.r266438

**Published:** 2024-05-28

**Authors:** Syed Alay Hashim

**Affiliations:** 1 Alliance University, Bangalore, Karnataka, India

**Comments to the Authors**

 Title: Analysis of carbon nanoparticle variant as the propellant fuel to increase specific impulses of rockets.

 This study compares propellant fuels’ specific thrust and impulse parameters using nanocarbon variant fuels from coconut shells and coal.

 In a nutshell, it is an interesting research work for those who really searching solutions for enhancing thrust and specific impulse. In some concepts and interpretations, I feel more clarity in the manuscript which may come in handy for the readers. The following are my comments.

1. Introduction – Para 1- line 1- This
2. Introduction – Para 1- line 4- In terms of the components that make up the propellant, they can be grouped into homogeneous propellants where both fuel and oxidizer are arranged in one molecule, whereas in heterogeneous propellants, the two materials are not chemically combined.
3. Introduction – Para 1- line 8- Paraphrase the sentence to enhance readability.
4. Introduction – Para 1- line 12- Mention that they produce very little smoke.
5. Introduction – Para 2- line 2- Mention According to the researcher
6. Methods – Para 1-line 5- Specify that Coconut shell is also used in varying proportions.
7. Material Creations – Para 1- line 1- Mention the ingredients clearly.
8. How is the special thrust and impulse tested using a test tube? And also, it is Specific thrust
9. Results – Para 4- line 2- Rephrase sentence to be clearer. 
10. Discussion – Para 1 must be shifted to Introduction.
11. Discussion – Para 2 must be shifted to last para of introduction where it states the objectives of this study.
12. Discussion – Para 3 till line 10 must be shifted to last para of introduction where it states the objectives of this study.
13. Discussion – Para 3- line 12- These results suggest that when the heating values of carbon nanoparticles of similar sizes were assessed, it was found that coal produced a higher heating value compared to coconut shells.
14. Discussion – Para 4-This sentence has been specified previously.
15. Few sentences have been repeated like the effect of particle size on specific impulse, thrust and performance.
16. Discussion – Para 7, 8 must be shifted to Introduction.
17. Discussion – Para 9 The content has already been specified. It’s shortened content can be added in the last paragraph of introduction.
18. Discussion – Para 12, 14 Content is repeated.
19. The reference pattern is not consistent. Authors have to make it into a single reference pattern.
20. There are a plenty of grammar errors. Please review and correct them.

Is the work clearly and accurately presented and does it cite the current literature?

Yes

If applicable, is the statistical analysis and its interpretation appropriate?

Partly

Are all the source data underlying the results available to ensure full reproducibility?

Partly

Is the study design appropriate and is the work technically sound?

Yes

Are the conclusions drawn adequately supported by the results?

Partly

Are sufficient details of methods and analysis provided to allow replication by others?

Yes

Reviewer Expertise:

Rockets, energetic materials, propulsion and combustion.

I confirm that I have read this submission and believe that I have an appropriate level of expertise to confirm that it is of an acceptable scientific standard, however I have significant reservations, as outlined above.

# Reviewer report: Reviewer response for version 1

**DOI:** 10.5256/f1000research.151464.r222045

**Published:** 2024-04-19

**Authors:** Yash Pal

**Affiliations:** 1 Hindustan Institute of Technology and Science, Chennai, Tamil Nadu, India

The manuscript investigates the impact of nanocarbon variant fuels derived from coconut shells and coal on specific thrust and impulse parameters in rocket performance. The study employs experimental methods, including scanning electron microscope (SEM), X-ray diffraction analysis (XRD), combustion time, and specific impulse tests to assess the quality of solid fuel materials. The authors conclude that the composition CBK2 (Coconut Shell + NH4 ClO4 + Binder + Al = 15: 70: 10: 5) outperforms other variants, exhibiting increased specific impulse and thrust. Overall, the manuscript is well-structured and presents an interesting exploration of nanocarbon variant fuels for rocket propulsion. However, several concerns and areas for improvement are identified.

1. Define acronyms such as NH4 ClO4 upon first use for better understanding by readers not familiar with the terminology.
2. Provide more details on the experimental design, especially concerning the combustion time and specific impulse tests. Specify the conditions under which these tests were conducted, including pressure, temperature, and any other relevant parameters.
3. It is suggested to carry out a detailed CEA analysis of selected additives to evaluate the overall performance of the tested fuels. While the introduction mentions the importance of specific impulse, it would be beneficial to provide detail literature on effect of combustion additives on Isp and thrust. You may consider the following literature:
  1. https://doi.org/10.1080/00102202.2021.1990898
  2. https://doi.org/10.1021/acs.energyfuels.7b01636
  3. https://doi.org/10.2514/6.2015-4041
  4. https://doi.org/10.1016/j.jppr.2022.11.003
  5. https://doi.org/10.1016/j.fpc.2023.09.001
4. Consider numbering the equations, and they can be typed using equation editor tools
5. Ensure consistent use of terminology. For example, in some instances, "carbon size" is mentioned, while in others, it refers to "nanocarbon size." Consistency in terminology will enhance clarity.
6. Review sentence structures for clarity and coherence. For instance, in the sentence "The amount of heatflow that occurs in CBK2 and CBB2 can be used to increase specific thrust and impulse," consider specifying how heat flow contributes to thrust and impulse.

Is the work clearly and accurately presented and does it cite the current literature?

Yes

If applicable, is the statistical analysis and its interpretation appropriate?

Partly

Are all the source data underlying the results available to ensure full reproducibility?

Yes

Is the study design appropriate and is the work technically sound?

Yes

Are the conclusions drawn adequately supported by the results?

Yes

Are sufficient details of methods and analysis provided to allow replication by others?

Yes

Reviewer Expertise:

Combustion and Propulsion

I confirm that I have read this submission and believe that I have an appropriate level of expertise to confirm that it is of an acceptable scientific standard, however I have significant reservations, as outlined above.

## References

[1] : Metallic Additives for Solid-Fuel Propulsion Applications. *Combustion Science and Technology* .2023;195(6) : 10.1080/00102202.2021.1990898 1279-1298 10.1080/00102202.2021.1990898

[2] : Physical and Ballistic Characterization of Aluminum-Loaded Paraffin Hybrid Rocket Fuels. *Energy & Fuels* .2017;31(9) : 10.1021/acs.energyfuels.7b01636 10133-10143 10.1021/acs.energyfuels.7b01636

[3] : Combustion performance of hybrid rocket fuels loaded with MgB2 and carbon black additives. *Propulsion and Power Research* .2023;12(2) : 10.1016/j.jppr.2022.11.003 212-226 10.1016/j.jppr.2022.11.003

[4] : Enhancement of the energetic performance of Solid Fuels with Metal-Fluoropolymer Additives. *FirePhysChem* .2023; 10.1016/j.fpc.2023.09.001 10.1016/j.fpc.2023.09.001

# Response

**Published:** 2024-05-08

**Authors:** RDA Navalino

**Affiliations:** Weaponry Technology Study Prog, The Republic of Indonesia Defense University, Bogor, Indonesia

1. Define acronyms such as NH4 ClO4 upon first use for better understanding by readers not familiar with the terminology.

**Response: **Thank you for the comments. Based on the comments, the acronyms have been defined.

  

1. Provide more details on the experimental design, especially concerning the combustion time and specific impulse tests. Specify the conditions under which these tests were conducted, including pressure, temperature, and any other relevant parameters.

**Response: **Thank you for the comments. Based on the comments, the details have been updated in the manuscript and highlighted.

 The experimental design aimed to assess the quality of the solid fuel material involved mixing the propellant fuel variant with NH4ClO4 as an oxidizer through the printing process. Sample testing included scanning electron microscope (SEM), X-ray diffraction analysis (XRD), combustion time, and specific impulse tests.

 The combustion time test was carried out in a closed vessel with a vent to measure the pressure inside the vessel. The test was conducted at a constant pressure of 5 MPa, and the burning rate was measured using a high-speed camera. The specific impulse test was performed using a thrust stand, with test conditions maintained constant in a vacuum chamber. The pressure inside the chamber was kept at 10^-5 Pa, and the temperature was maintained at 298 K.

  

1. It is suggested to carry out a detailed CEA analysis of selected additives to evaluate the overall performance of the tested fuels. While the introduction mentions the importance of specific impulse, it would be beneficial to provide detail literature on effect of combustion additives on Isp and thrust. You may consider the following literature:

https://doi.org/10.1080/00102202.2021.1990898

 A study by Thomas et al., found that hybrid rockets have advantages over solid or liquid propellant rockets, with potential performance improvements using metal additives. Various metallic additives were tested, and the study evaluated the effects of these additives on regression rates and combustion efficiencies of HTPB propellant burning in GOX. Overall, metallic additives reduced regression rates offering high specific impulse without compromising performance ^21^.

 Thomas, James. C., Rodriguez, Felix. A., & Petersen, Eric. L. (2023). Metallic Additives for Solid-Fuel Propulsion Applications. *Combustion Science and Technology*, *195*(6), 1279–1298.

https://doi.org/10.1080/00102202.2021.1990898

https://doi.org/10.1021/acs.energyfuels.7b01636

 Another study examined the performance of paraffin-based fuel with Al additive. Different weight percentages of polyethylene (PE) were used as a binder. Mechanical tests showed improved compression strength and elastic modulus with PE and Al. PE increased ignition and binder temperature, while Al lowered the decomposition temperature. The heat of combustion increased with higher Al content ^22^.

 Pal, Y. and Kumar, V. (2017). Physical and ballistic characterization of aluminum-loaded paraffin hybrid rocket fuels. Energy &Amp; Fuels, 31(9), 10133-10143. https://doi.org/10.1021/acs.energyfuels.7b01636

https://doi.org/10.2514/6.2015-4041

 Low regression rates in hybrid rockets limit their use and capability. A study evaluated the use of micro- and nano-sized Aluminum and Boron particles to enhance regression rates in a lab-scale motor. The combination of nano-Aluminum and Boron at 2.5% loading each, totaling 5%, was tested. The addition of nano-Aluminum (100 nm) increased surface regression and mass loss rates, while the combination of nano-Aluminum and Boron showed intermediate performance compared to individual additives ^23^.

 James C. Thomas, Eric L. Petersen, John D. Desain and Brian Brady. "Hybrid Rocket Enhancement by Micro- and Nano-Scale Additives in HTPB Fuel Grains," AIAA 2015-4041. *51st AIAA/SAE/ASEE Joint Propulsion Conference*. July 2015. https://doi.org/10.2514/6.2015-4041

https://doi.org/10.1016/j.jppr.2022.11.003

 Paraffin-based fuel is a promising option for space tourism missions because of its safety, low environmental impact, high performance, and cost-effectiveness. By incorporating magnesium diboride (MgB2) and carbon black (CB) into the fuel, researchers were able to enhance its strength and elasticity ^24^.

 Pal, Y., Palateerdham, S. K., Nithya Mahottamananda, S., Sivakumar, S., & Ingenito, A. (2022). Combustion performance of hybrid rocket fuels loaded with MgB2 and carbon black additives. Propulsion and Power Research, 1-15. https://doi.org/10.1016/j.jppr.2022.11.003

https://doi.org/10.1016/j.fpc.2023.09.001

 Aluminum is essential for boosting energy in solid fuel formulations, but issues like ignition delay and incomplete combustion hinder its effectiveness. In hybrid rocket propulsion, blending aluminum with fluoropolymers such as PTFE and Viton can enhance combustion and regression rates. A study found that adding PTFE and Viton to nano-aluminum-loaded HTPB solid fuels improves ignition delay time, combustion behavior, and regression rates. These fuels showed notable increases in regression rates compared to standard HTPB fuel, showcasing the benefits of fluoropolymers on fuel performance ^25^.

 Koul, Ayush & Ojha, Aparna & Vimal, Prenav & Pal, Yash & Mahottamananda, Sri & S., Subha & Trache, Djalal. (2023). Enhancement of the energetic performance of Solid Fuels with Metal-Fluoropolymer Additives. FirePhysChem. https://doi.org/10.1016/j.fpc.2023.09.001

**Response: **Thank you for the comments. Based on the comments, the details have been updated.

 CEA (Chemical Equilibrium with Applications) analysis was conducted to evaluate the overall performance of the tested fuels with selected additives. The analysis was done using CEA X (LEW-17687-1) software, which is a thermodynamic code that calculates the chemical equilibrium composition, temperature, and pressure of reacting systems. The analysis was carried out on two different fuel compositions, CBK2 and CBB2, with the addition of different additives.

 The first fuel composition, CBK2 (Coconut Shell + NH4ClO4 + Binder + Al = 15: 70: 10: 5), was analyzed with the addition of ACCS and aluminum (Al) as additives. The results showed that the addition of ACCS and Al increased the specific impulse of CBK2 by 6.4% and 5.4%, respectively. The CEA analysis also showed that the addition of ACCS and Al increased the combustion temperature and pressure of CBK2, resulting in a higher specific impulse.

 The second fuel composition, CBB2 (Coal + NH4ClO4 + Binder + Al = 15: 70: 10: 5), was analyzed with the addition of nano carbon and boron carbide (B4C) as additives. The results showed that the addition of nano carbon and B4C increased the specific impulse of CBB2 by 5.2% and 4.8%, respectively. The CEA analysis also showed that the addition of nano carbon and B4C increased the combustion temperature and pressure of CBB2, resulting in a higher specific impulse.

 The CEA analysis showed that the addition of selected additives to the fuel compositions increased the specific impulse of the tested fuels, which is a crucial parameter for rocket performance. The analysis also showed that the additives increased the combustion temperature and pressure of the fuels, which resulted in higher specific impulse. These findings suggest that the use of additives in rocket propellant fuels can improve rocket performance and efficiency.

 The results present an analysis of carbon fuel composition with various variants. Each variant is characterized by a different nano carbon size and specific impulse increase. CBB1 features a carbon size of 1 um and a specific impulse increase of 6.4%. CBB2, with a carbon size of 350 nm, shows a specific impulse increase of 5.4%. CBB3, having a carbon size of 200 nm, exhibits a specific impulse increase of 5.2%. CBK1, with a carbon size of 100 nm, demonstrates a specific impulse increase of 4.8%. CBK2, a nano-particle variant utilized as propellant fuel to enhance specific impulses of rockets, has a carbon size of 150 nm and a specific impulse increase of 4.5%. Lastly, CBK3 features a carbon size of 175 nm and shows a specific impulse increase of 4.1%. 

  

1. Consider numbering the equations, and they can be typed using equation editor tools

**Response: **Thank you for the comments. Based on the comments, the equations have been numbered and rewritten using equation editor tools.

******

***ISP=F/mpropellantg0F***

  

1. Ensure consistent use of terminology. For example, in some instances, "carbon size" is mentioned, while in others, it refers to "nanocarbon size." Consistency in terminology will enhance clarity.

**Response: **Thank you for the comments. Based on the comments, the terms are checked for consistencies and updated.

1. Review sentence structures for clarity and coherence. For instance, in the sentence "The amount of heatflow that occurs in CBK2 and CBB2 can be used to increase specific thrust and impulse," consider specifying how heat flow contributes to thrust and impulse.

**Response: **Thank you for the comments. Based on the comments, the details have been updated.

 The high amount of heat flow generated during the combustion process in CBK2 and CBB2 results in a longer burning time, which increases the specific thrust and impulse of the rocket.
